# Prenatal heat stress effects on gestation and postnatal behavior in kid goats

**DOI:** 10.1371/journal.pone.0220221

**Published:** 2020-02-10

**Authors:** Wellington Coloma-García, Nabil Mehaba, Pol Llonch, Gerardo Caja, Xavier Such, Ahmed A. K. Salama

**Affiliations:** 1 Group of Research in Ruminants (G2R), Department of Animal and Food Science, Universitat Autònoma de Barcelona (UAB), Bellaterra, Barcelona, Spain; 2 Facultad de Medicina Veterinaria, Universidad Agraria del Ecuador (UAE), Guayaquil, Ecuador; 3 Service of Nutrition and Animal Welfare (SNiBA), Department of Animal and Food Science, Universitat Autònoma de Barcelona (UAB), Bellaterra, Barcelona, Spain; INIA, SPAIN

## Abstract

Consequences of heat stress during pregnancy can affect the normal development of the offspring. In the present experiment, 30 Murciano-Granadina dairy goats (41.8 ± 5.7 kg) were exposed to 2 thermal environments varying in temperature-humidity index (THI) from 12 days before mating to 45 days of gestation. The environmental conditions were: gestation under thermal-neutral (TN; THI = 71 ± 3); and gestation under heat stress (HS; THI = 85 ± 3) conditions. At 27 ± 4 days old, female kids exposed to *in utero* TN (IUTN; n = 16) or *in utero* HS (IUHS; n = 10) were subjected to 2 tests: arena test (AT) and novel object test (NOT), the latter was repeated at 3 months of age. Additionally, 8 months after birth, a subset of IUTH and IUHS growing goats (n = 8 each; 16.8 ± 3.4 kg BW) were exposed to 2 environmental conditions in 2 consecutive periods: a basal thermal-neutral period (THI = 72 ± 3) for 7 days, and a heat-stress period (THI = 87 ± 2) for 21 days. In both periods, feeding, resting, posture, and thermally-associated behaviors were recorded. The gestation length was shortened by 3 days in GHS goats. In the AT, IUHS kids showed a lower number of sniffs (*P* < 0.01) compared to IUTN. In the NOT, IUHS kids also tended to show a lower number of sniffs (*P* = 0.09). During heat exposure, IUTN and IUHS growing goats spent more time resting and exhibited more heat-stress related behaviors such as panting and drinking (*P* < 0.001); however, no differences were observed between both groups. In conclusion, heat stress during the first third of pregnancy shortened gestation length and influenced the exploratory behavior of the kids in the early life. However, behavior responses to heat stress during the adulthood were not affected by the *in utero* thermal treatment.

## Introduction

There is evidence that environmental conditions during pregnancy can modify fetal programming through physiological and epigenetic changes [1, 2], which permanently modify the behavior, health and productivity of the offspring. Several studies have shown that episodes of stress during the prenatal stage have negative effects on the pregnancy itself, by shortening its duration [3, 4], and on the postnatal life of the offspring by reducing birth weight [2].

Beyond these effects, maternal stress during pregnancy has shown to have profound effects on the development and function of the hypothalamic-pituitary-adrenal (HPA) axis, and the associated circulating ACTH and cortisol concentrations [5]. Moreover, recent research suggests that these effects remain in further generations [6]. In this regard, most studies using rodent or primate models show that gestational stress results in increased aggressiveness and altered social interactions [7–9] as well as a reduction in the neuromotor capacities, and exploration and learning [4,10].

In the future, as global warming progresses, an increase in temperatures accompanied by increasingly frequent heat waves is expected [11]. In the case of ruminants, heat stress during pregnancy has attracted special attention, due to the significant impact on food production (i.e. milk) [12]. Furthermore, although literature is scarce, thermal stress during pregnancy is demonstrated to be responsible for the abnormal development of the fetus and cause a harmful effect in the early postpartum period and adulthood. For instance, prenatal heat stress can impair the normal postnatal growth of the offspring, compromise the passive immunity, and also alter the behavioral patterns [13,14]. Nevertheless, the previous studies evaluated the impact of maternal heat stress during the late gestation in cows, but little is known about the effects of heat stress during early pregnancy on offspring behavior in dairy animals, including cows and goats. There is strong evidence that fetal programming occurs during early gestation in ruminants, and several environmental and nutritional factors during this period can condition performance of offspring permanently. For instance, adequate maternal nutrition in early gestation is critical for the normal development of all fetal organs and tissues [15]. Additionally, exposure of cows to limited nutrition during early gestation resulted in decreased skeletal muscle mass and altered glucose metabolism of offspring [16]. Therefore, we hypothesized that heat stress (with its related effects such as altered blood flow, changes in hormone levels, reduced feed intake, etc.) during early gestation would alter performance and response of offspring to environmental stimuli.

Besides the genetic component, environment is extremely important in shaping the animal behavior [17]. One of the first changes that can be observed in animals that are under stressful conditions is a change in their behavior repertoire. Within behavior, the way animals react to novel situations is also influenced by the environmental conditions in where they live [18]. Therefore, behavior is a sensitive measure to investigate changes of perception of the environment. The objective of the present study was to investigate the effect of heat stress in goats at the beginning of the pregnancy on the gestation performance of dams and the changes in the behavior of the offspring both at neonatal and adult stages.

## Materials and methods

The animal care conditions, treatment, housing, and management practices was approved by the Ethical Committee of Animals and Humans Experimentation of the UAB (Reference# 4790) and followed the procedures stated by the EU legislation (Regulation 2010/63/EC).

### Treatments and management conditions of dams

Thirty multiparous lactating Murciano-Granadina dairy goats of 41.8 ± 5.7 kg body weight (BW) and 4.9 ± 0.4 years of age from the experimental farm of the UAB were used. Goats were housed in 6 pens (5 × 2.5 m^2^) of 5 goats each, distributed equally in 2 adjacent rooms, one for each treatment. Goats were distributed by similar BW within each pen. The present experiment was carried out during spring (March to June). After 2 weeks of adaptation to the experimental conditions, goats were distributed in 2 groups exposed to 2 different climatic conditions (n = 15) from day 12 before mating until day 45 of gestation. The climatic conditions were: thermo-neutral (TN), and heat stress conditions (HS). The TN group was maintained at 15 and 20°C and 49 ± 8% relative humidity (temperature humidity index, THI = 71 ± 3, calculated according to NRC [19]), and HS group for 12-h day at 37 ± 0.5°C and 45 ± 5% relative humidity (THI = 86 ± 3) and 12-h night at 30 ± 0.5°C and 47 ± 2% relative humidity (THI = 78 ± 2). The temperature of the TN group was maintained with the help of one 3.5-kW electric heater equipped with a thermostat (General Electric, Barcelona, Spain) when necessary. The room housing HS animals was equipped with four 3.5-kW electric heaters coupled to thermostat (General Electric). Environmental temperature and humidity were recorded every 10 min throughout the experiment by data loggers (Opus 10, Lufft, Fellbach, Germany). Both treatments were maintained from 12 days before mating until 45 days after mating (early gestation).

Goats were mated in April, and they were divided into 6 mating groups (5 goats each), resulting in 3 TN and 3 HS groups. Estrus was synchronized in a way that 2 to 3-day intervals were allowed between each mating group. Synchronization was performed using intravaginal sponges (progesterone P4; Sincropart 30 mg, Ceva Animal Health, Barcelona, Spain) for 12 days followed by the administration of equine-chorionic gonadotropin (eCG, 400 IU; Ceva Animal Health) at the time of sponge withdrawal. The 6-goat groups were naturally mated by the same buck at 2 to 3-day intervals. On the day of mating, the buck served the 5 goats twice in the morning and afternoon. For that purpose, each goat was taken outside the chamber to a raceway and introduced to the buck. Consequently, the male had 2 to 3-d rest periods between mating groups. Pregnancy was confirmed by transrectal ultrasound at days 21 and 45 after mating, and all goats were confirmed to be pregnant.

Feed was provided *ad libitum* as a total mixed ration (70% alfalfa hay and 30% concentrate). Concentrate contained barley 31.5%, corn 41.5%, soybean meal 44.5%, sodium bicarbonate 1%, calcium phosphate 0.4%, calcium carbonate 0.5%, salt 0.7%, and premix 0.4%; as fed basis. Water was freely available at room temperature. Mineral salt blocks (Na 36.7%, Ca 0.32%, Mg 1.09%, Zn 5 g/kg, Mn 1.5g /kg, S 912 mg/kg, Fe 304 mg/kg, I 75 mg/kg, Co 50 mg/kg, and Se 25 mg/kg; Ovi Bloc, Sal Cupido, Terrassa, Spain) were freely available in each pen throughout the experiment.

Goats were milked twice per day using a mobile milking unit set at 42 kPa, 90 pulses/min, and 66% pulsation ratio. Feed intake was recorded daily, calculated by the difference between the weight of the ration offered and the leftover at the end of the day. Rectal temperature (RT) and respiration rate (RR) were recorded daily 3 times per day at 8, 12, and 17 h. RT was recorded with a digital veterinary thermometer (ST714AC Accu-vet, Tecnovet S.L, Barcelona, Spain). RR was calculated as the number of breaths per minute by counting the flank movements with the help of a chronograph and from a distance of 2 m without disturbing the goats.

**Fig 1:**
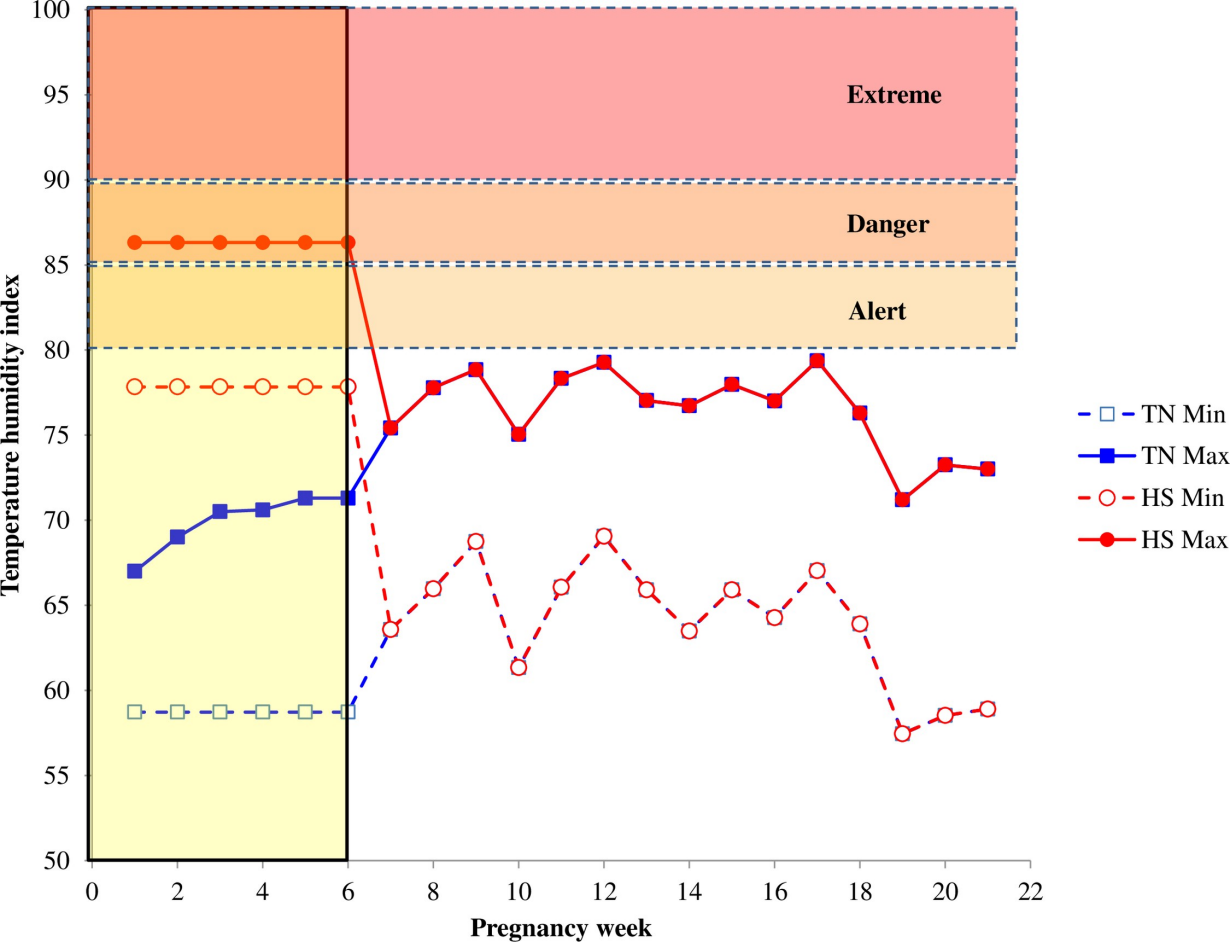
Minimum and maximum THI values throughout the experiment. **The vertical shaded strip indicates when goats were in 2 climatic chambers under thermal-neutral (TN) and heat stress (HS) conditions. Afterwards, both goat groups were moved to the barn under semi-extensitve conditions.** Levels of heat stress (alert, danger, and extreme) are indicated by the horizontal shaded strips according to Silanikove and Koluman [20]. For the first 6 weeks of pregnancy, HS-goats were in the danger level only during the day time and decended to the normal level during night. When goats were moved to the barn the maximum THI values were in the upper part of the normal level. The THI values were calculated using temperature and humidity data recorded by the data loger when goats were in the climatic chambers, and by the data provided by the closest meteorological ststion when they were in the barn.

After 45 days of gestation, all goats were gathered in one group and managed in the same barn under semi-intensive conditions (grazing 6 h/day and feed complemented when indoors). The minimum and maximum THI values recorded when goats were in the climatic chambers and when they moved to the barn until kidding are shown in Fig 1. Two weeks before the expected date of parturition, the goats were weighed and moved to kidding pens for permanent surveillance and parturition assistance. Immediately after birth, kids were separated from the goats and fed with their mothers’ colostrum and reared together with milk replacer (150 g/L, Elvor, Saint-Brice, France) with an automatic milk provider (Foerster-technik, Engen, Germany). Pregnancy length and litter size of kids were recorded after parturition. The BW of kids was recorded at birth and every week until 5 weeks old with a digital scale (Tru-Test AG500 Digital Indicator, accuracy, Auckland, New Zealand).

### Behavioral tests and measurements on female kids

For the behavioral assessment, female kids at 27 ± 4 days old, from IUTN (n = 16) and IUHS (n = 10) groups were individually exposed to an arena test (AT) for 5 consecutive days, and to a novel object test (NOT) at 48 h after the end of the AT. The NOT test was repeated at 3 months of age. The AT and NOT were carried out in an anechoic climatic chamber (Eurosheild, ETS Lindgren-Euroshield Oy, Eura, Finland) in order to avoid sounds from outside and variations of temperature. Both AT and NOT were video recorded for subsequent analysis. Furthermore, behavioral response to a heat challenge was evaluated at 8 months of age using 16 female growing goats (8 IUTN and 8 IUHS).

#### Arena test (AT)

The AT was carried out in a 4 × 4 × 2.3 m^3^ arena (w × l × h), in which 9 squares of 1.3 × 1.3 m^2^ were painted on the ground with chalk. The access to the arena was through a starting cage of 50 × 50 × 60 cm^3^ (w × l × h) separated from the arena by a guillotine door (S1A Fig). On the test day, each kid was randomly selected among the 2 treatments, moved to the starting cage and freed 30 s later into the arena. The duration of the test was 8 min and time started to run when the kid was completely inside the arena. The following behavioral parameters were measured: number of squares entered, frequency of jumping and sniffing (nose less than 5 cm from the walls or floor) events, number of vocalizations and time spent moving forward [21].

#### Novel object test (NOT)

For NOT the same procedure was followed as for AT and the same behavioral measurements were registered. In addition, a road hazard cone (0.5 × 0.7 m^2^, w × h) was placed on the floor against the wall opposite to the starting cage (S1B Fig); thereby the latency and the frequency of sniffing events addressed to the novel object were registered [21]. The NOT test was repeated at 3 months of age.

### Heat stress challenge test

To compare the behavioral response of IUTN and IUHS animals to the same stressor (i.e., heat stress) after sexual maturity, a subset of the growing goats was selected at 8 months of age. Kids were born in September and the heat stress test was done in May; thus both IUTN and IUHS goats did not experience summer conditions before the test. The IUTN and IUHS goats were balanced by BW and mother parity, and randomly allocated to individual pens (1.08 m^2^) with 8 replicates per group in the same room. After one week for adaptation to facilities, 2 different climatic conditions were applied in 2 consecutive periods to both groups. During the first period, basal TN period (1 week), temperature and humidity averaged 24 ± 2.43°C and 68 ± 9% (THI = 72), respectively. During the second period (3 weeks), goats were exposed to HS conditions, where the average temperature was 37 ± 1.8°C and humidity was 49 ± 7.0% (THI = 87) during the day and 31 ± 1.4°C and 53 ± 7.0% (THI = 80), respectively, at night. Room temperature was automatically controlled by 4 electric heaters with a thermostat (3.5 kW; General Electric). Environmental temperature and humidity were continuously recorded every 10 min throughout the experiment by data loggers (Opus 10, Lufft, Fellbach, Germany).

Feed was provided as a total mixed ration consisting of 85% alfalfa hay and 15% concentrate (as feed basis: oat grain 5%, malting barley 10%, canola meal 10%, gluten feed 10%, corn 4.7%, soy hulls 45%, soybean oil 5%, soybean meal 5%, molasses 2%, bicalcic phosphate 2.5%, salt 0.5%, premix 0.3%) once daily at 9:30 h. Clean water was freely and individually available for each goat.

A single trained observer recorded behavior following a scan-sampling methodology [22]. Behaviors were recorded between 12 h and 17 h, within the period of heat stress. The behavioral observations were performed daily and the duration of each session was 2 h, whereby each pen was scanned 40 times at 3 min interval.

The behavioral measurements were drawn from the Welfare Assessment Protocol for Goats [23]. Feeding (feeding + rumination + drinking), other non-feeding active and inactive behaviors (exploration + grooming + other + resting) and physiological behavior associated to thermal stress (open-mouth or close-mouth panting) were recorded as well as posture (standing-walking + standing-immobile + lying-straight + lying-joint). The definition of the recorded behaviors is shown in S1 Table.

### Statistical analyses

The duration of pregnancy and birth weight were analyzed with the GLM procedure of SAS (version 9.4; SAS Institute Inc., Cary, NC). The feed intake, RT and RR measurements on goats (dams) were analyzed as repeated measures using a linear mixed model (PROC MIXED procedure of SAS). Behavioral data from NOT and scan sampling during the heat exposure test, as counts and week average percentages, respectively, were analyzed as repeated measures using a generalized linear mixed occasional behaviors, using a generalized linear model (PROC GENMOD), all adjusted under a Poisson or a Negative Binomial distribution, according to the fitness of the model. Also, litter size was analyzed using the PROC GENMOD procedure. The models included treatment (HS vs TN for dams and IUHS vs IUTN for kids) as fixed effect, and in the case of repeated measures, day or week was also included as a fixed effect as well as the interaction of treatment × day or treatment × week, while animal was considered as a random effect. Litter size was used as a covariable for the analysis of the duration of pregnancy. For litter weight, the effects treatment, sex, litter size, and treatment × sex interaction were considered in the model. Differences between least squares means were determined with the PDIFF test of SAS. Significance was declared at *P* < 0.05 and trend at *P* < 0.10 unless otherwise indicated.

## Results

### Effects of heat stress during the pregnancy and early postpartum

Regarding the physiology measurements of goats during the experimental period, HS goats showed a greater (*P* < 0.01) RT compared to TN goats (average 38.7°C for TN and 39.3°C for HS; SED = 0.07) and greater (*P* < 0.01) RR (average 33 breaths/min for TN and 108 breaths/min for HS; SED = 3.06), Feed intake was lower (*P* < 0.01) in HS compared to TN goats (2.52 kg/day for TN and 2.12 kg/day for HS; SED = 0.55).

The results of the different variables evaluated at parturition and early postpartum period are shown in Table 1. Although HS goats in the present study were mated under high ambient temperatures, both TN and HS goats were effectively fertilized as indicated by the transrectal ultrasonography at days 21 and 45 of pregnancy. However, 4 goats (2 TN and 2 HS) did not deliver kids. These 4 goats could have suffered fetus losses after the pregnancy diagnosis has been done. The gestation length was on average shortened by 3 days in HS goats compared to TN (*P* = 0.006). Thirty and 29 kids were born to TN and HS goats, respectively. The litter weight of HS group tended (*P* = 0.061) to be lower for HS compared to TN goats despite the fact that more male kids were born to HS (n = 13) than TN (n = 7) goats, and males weighed more than females (2.50 ± 0.10 vs. 2.37 ± 0.07 kg for males and females, respectively; *P* < 0.05). Litter weight was influenced by the litter size (*P* < 0.001), as a greater litter size was associated to smaller kids. However, kids’ body weight at 35 days of age was not affected by the treatment (*P* > 0.10). No kids were born dead. However, 4 (1 male and 3 females) and 6 (3 males and 3 females) kids were died at the age of 9 ± 2 days from TN and HS groups, respectively. Reasons for mortality included pneumonia, clostridium diarrhea, and accidental crush.

**Table 1:** Gestation length in dams and performance of kids at birth and early postpartum period.

Item	Treatment^1^		SED^2^	Effect (*P*-value)	
	TN	HS		Treatment	Litter size^3^
Litter size	2.31	2.23	0.31	0.806	-
Litter weight, kg	5.40	4.71	0.71	0.061	0.001
Duration of pregnancy, day	146	143	0.9	0.006	0.915
Birth-weight of kids^4^, kg	2.34	2.18	0.10	0.122	-
Weight of 35-days-old kids^5^, kg	7.88	7.64	0.54	0.520	-

^1^ TN, dams exposed to thermal-neutral conditions during the first 45 days of gestation (n = 15); HS, dams exposed to heat-stress during the first 45 days of gestation (n = 15).

^2^ Standard error of the difference.

^3^ Litter size used as a covariable.

^4^ n = 30 kids for TN, and n = 29 for HS.

^5^ n = 26 kids for TN, and n = 23 for HS.

### Behavioral responses to arena test

The results of the behavioral responses to the AT test are summarized in Table 2. A significant day effect was observed for all behavior variables, as the number of vocalizations (*P* < 0.001) and time spent moving forward (*P* = 0.001) decreased, whereas the number of jumping (*P* < 0.001) and sniffing events (*P* = 0.009) increased from day 1 to day 5, reflecting familiarization of kids with the arena test facilities. Further, the number of squares that kids walked through showed to be higher from day 1 to day 2 afterwards being diminished towards day 5 (*P* ≤ 0.001), which is consistent with the reduction in the time spent moving forward. Regarding the effect of treatment, IUHS kids showed a lower number of sniffing events compared to GTN kids (*P* = 0.009). Additionally, the significant interaction between treatment and day for vocalizations (*P* < 0.001) was due to the fact that the number of vocalizations in the IUHS kids was lower during the 2 first days (*P* ≤ 0.05) and recovered thereafter. The rest of behavioral parameters assessed were not influenced by the *in utero* thermal treatment (*P* > 0.10).

**Table 2:** Behavioral responses in arena test (AT) of female kids during 5 consecutive days.

Item	Treatment^1^		SED^2^	Effect (*P*-value)		
	IUTN	IUHS		Trt^3^	Day	Trt×Day
No. of squares entered	43.4	31.5	4.87	0.115	0.009	0.704
No. of jumps	1.54	1.15	0.48	0.586	0.001	0.546
No. of sniffs of the arena	33.5	26.7	1.62	0.007	0.001	0.335
No. of vocalizations	171	150	11.7	0.200	0.001	0.001
Time spent moving forward, s	54.8	44.9	7.06	0.282	0.001	0.123

^1^ IUTN, kids born to dams exposed to thermal-neutral conditions during the first 45 days of gestation (n = 16); IUHS, kids born to dams exposed to heat stress conditions during the first 45 days of gestation (n = 10).

^2^ Standard error of the difference.

^3^ Trt, treatment effect (IUHS vs. IUTN).

### Behavioral responses to novel object test

The novel object test (NOT) was performed at 1 and 3 months of age and results are shown in Table 3. At 1 month of age, IUHS kids showed trended to decrease the number of sniffing events compared to IUTN kids (*P* = 0.093), indicating a weaker motivation for exploration of novel objects in kids whose mothers suffered from heat stress during gestation. No treatment differences were detected for the other behavioral variables. At 3 months of age, no treatment effects were found on any of the variables assessed in the NOT.

**Table 3:** Behavioral responses in novel object test (NOT) of female kids at 1 and 3 months of age. Values are presented as means ± standard deviation.

Item	Treatment^1^		Effect (*P*-value)
	IUTN	IUHS	Treatment
**1 month of age**			
No. of squares entered	47.5 ± 1.08	38.9 ± 1.10	0.127
No. of jumps	4.81 ± 1.70	2.30 ± 1.98	0.413
No. of sniffs of the arena	36.1 ± 1.06	30.3 ± 1.08	0.093
No. of vocalizations	156 ± 1.1	162 ± 1.1	0.670
Time spent moving forward, s	48.8 ± 3.87	41.0 ± 4.90	0.220
No. of sniffs of the object	14.8 ± 1.14	10.5 ± 1.19	0.136
Latency before 1st sniff of the object, s	53.9 ± 35.8	77.4 ± 24.8	0.562
**3 months of age**			
No. of squares entered	41.3 ± 1.10	41.3 ± 1.13	0.998
No. of jumps	0.31 ± 1.56	0.50 ± 1.56	0.461
No. of sniffs of the arena	30.1 ± 1.06	33.5 ± 1.08	0.286
No. of vocalizations	168 ± 1.0	157 ± 1.0	0.670
Time spent moving forward, s	59.0 ± 6.46	53.0 ± 8.17	0.609
No. of sniffs of the object	5.25 ± 1.15	4.40 ± 1.22	0.136
Latency before 1st sniff of the object, s	43.2 ± 7.47	40.9 ± 9.96	0.855

^1^ IUTN, kids born to dams exposed to thermal-neutral conditions during the first 45 days of gestation (n = 16); IUHS, kids born to dams exposed to heat stress conditions during the first 45 days of gestation (n = 10).

### Behavioral responses to the heat challenge

The behavior variables measured at 8 months of age during the heat stress challenge are summarized in Table 4. No differences were observed between IUTN and IUHS goats for any of the variables measured (*P* > 0.10). Only lying-straight showed a treatment per time interaction trend (*P* = 0.099), however, no further differences were found between IUTN and IUHS animals neither during the basal TN period (1 week) nor during the heat-stress period (3 weeks).

**Table 4:** Behavioral and postural average expression of growing goats over the basal thermal neutral period and the heat stress challenge period.

Item	Treatment^1^		SED^2^	Effect (*P*-value)		
	IUTN	IUHS		Trt^3^	Week^4^	Trt×week
**Feeding behavior (%)**						
Feeding	24.0	25.2	2.1	0.702	0.001	0.533
Rumination	14.1	16.9	1.5	0.179	0.001	0.345
Drinking	2.02	1.58	0.40	0.042	0.001	0.857
**Non-feeding behaviors (%)**						
Exploration	4.46	4.88	0.81	0.709	0.001	0.231
Grooming	3.81	3.77	0.52	0.957	0.001	0.613
Other	3.07	2.77	0.44	0.645	0.003	0.390
Resting	41.2	37.9	2.35	0.312	0.001	0.361
**Thermally-associated behavior (%)**						
Open-mouth panting	0.99	1.18	0.63	0.786	0.001	0.989
Close-mouth panting	41.6	37.0	3.90	0.448	0.001	0.502
**Postures (%)**						
Standing-walking	1.38	1.55	1.55	0.644	0.001	0.985
Standing-immobile	33.6	35.3	2.64	0.643	0.001	0.718
Lying-joint	54.5	50.9	3.82	0.505	0.001	0.703
Lying-straight	4.95	5.48	2.17	0.859	0.001	0.099
Neck extended	0.42	0.23	0.23	0.736	0.006	0.249

^1^ IUTN, kids born to dams exposed to thermal-neutral conditions during the first 45 days of gestation (n = 8); IUHS, kids born to dams exposed to heat stress conditions during the first 45 days of gestation (n = 8).

^2^ Standard error of the difference.

^3^ Trt, treatment effect (IUHS vs. IUTN).

^4^ Basal period corresponded to the first week at thermal-neutral conditions and heat-stress (HS) period corresponded to the following three weeks.

**Fig 2:**
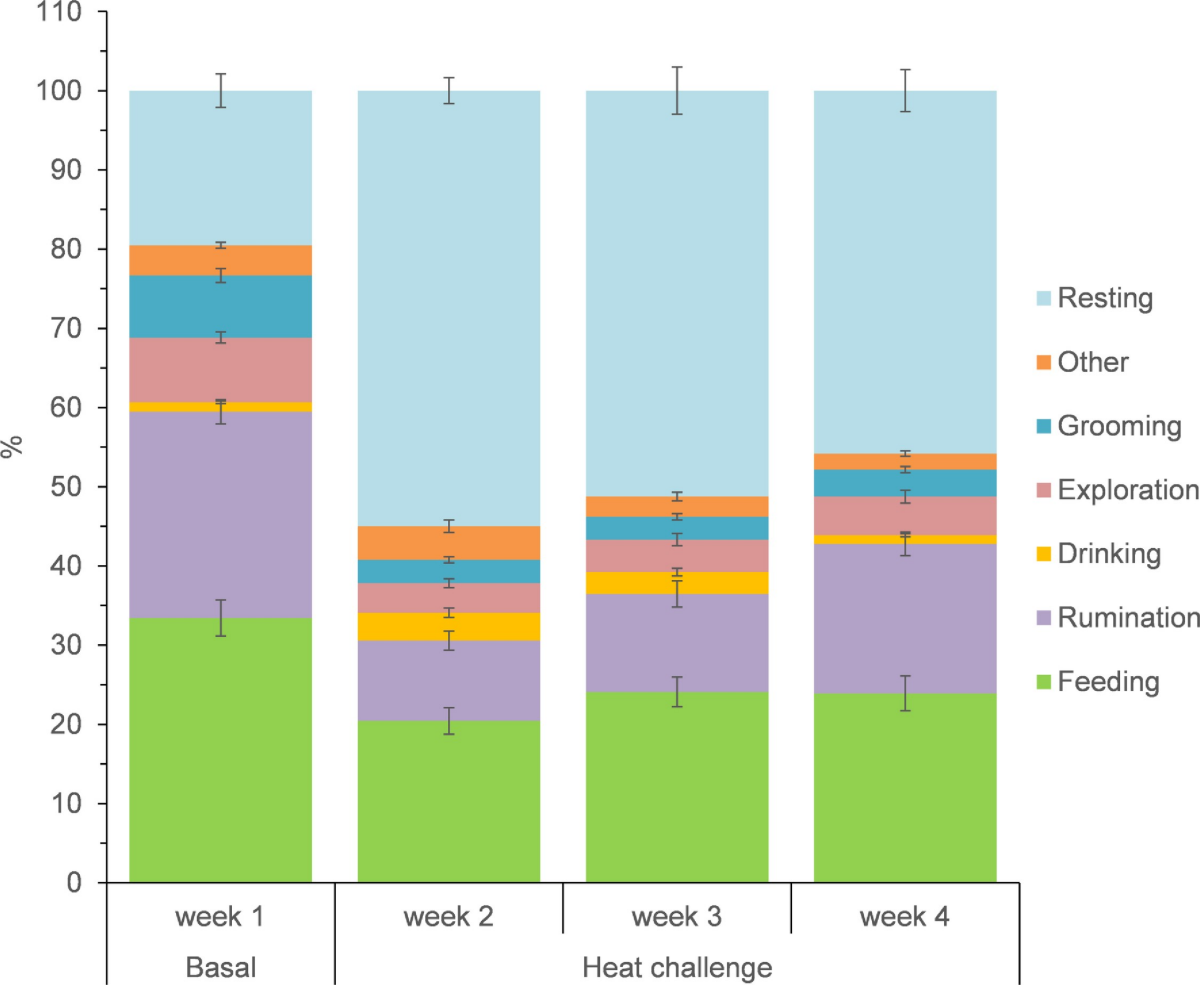
Activity behavior average expression (%) of growing goats over the basal thermal-neutral period (week 1) and during the heat-challenge period for 3 weeks (weeks 2 to 4). Bars indicate standard error.

All parameters were affected by the heat-stress challenge independent of the *in utero* thermal treatment as shown in Fig 2. Feeding, exploration and grooming behaviors were reduced immediately after the heat challenge (week 2) and remained low compared to the basal TN period (*P* < 0.001) in both, IUTN and IUHS goats. Rumination was also reduced during the heat challenge, but it started to recover towards the end of the experiment although never returned to the basal thermal-neutral values (week 4; *P* < 0.001). Drinking behavior also increased dramatically during the first week of exposure to heat (*P* < 0.001), but returned to initial values at the end of the experiment. Resting also increased progressively throughout the exposure to heat stress although did not reach basal values by the end of the experiment.

**Fig 3:**
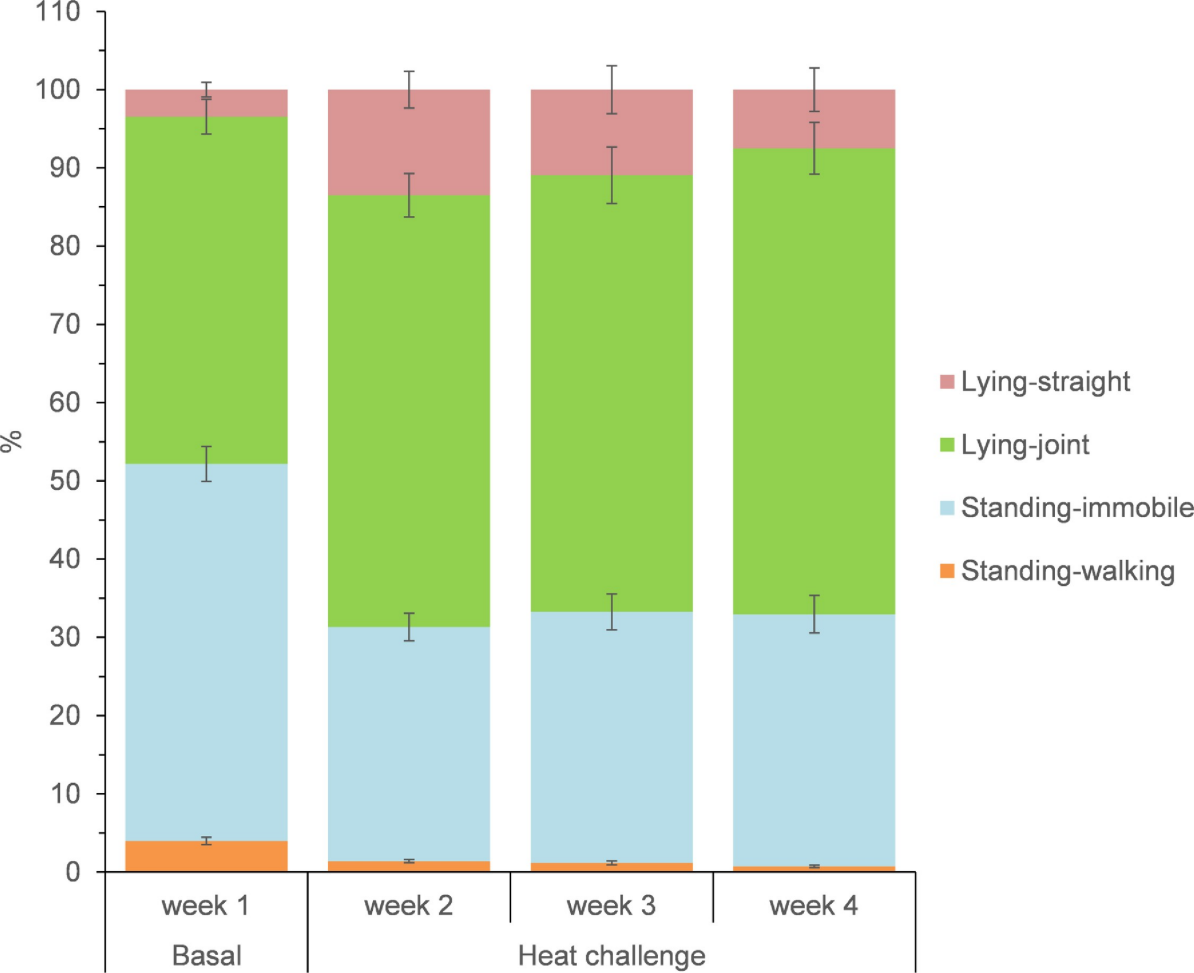
Posture average expression (%) of growing goats over the basal thermal-neutral period (week 1) and during the heat-challenge period for 3 weeks (weeks 2 to 4). Bars indicate standard error.

Postural behaviors averages are presented in Fig 3. Animals were lying more frequently during the heat challenge (*P* < 0.01), predominantly with legs joint, which resulted in less (*P* < 0.001) standing (immobile or walking) behaviors.

**Fig 4:**
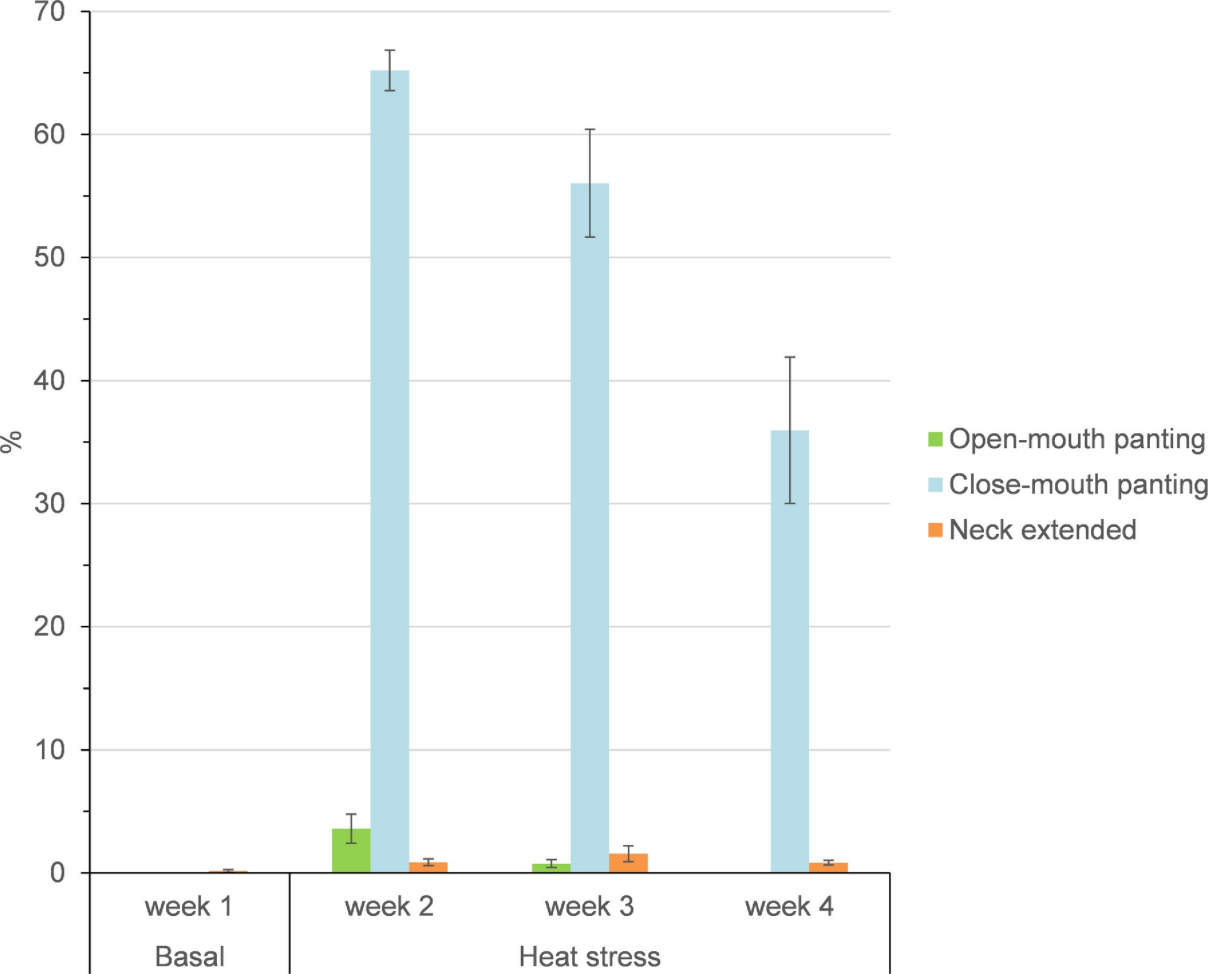
Thermally-associated behavior average expression (%) of growing goats over the basal thermal-neutral period (week 1) and during the heat-challenge period for 3 weeks (weeks 2 to 4). Bars indicate standard error.

As shown in Fig 4, the neck was extended more (*P* = 0.018) frequently during the heat challenge period compared to the basal TN week. Furthermore, goats experienced greater (*P* < 0.001) close-mouth panting after being exposed to heat and reduced (*P* < 0.05) this behavior progressively towards the end of the heat stress period. Open-mouth panting was highest at week 1 of HS and disappeared by week 3 of heat stress.

## Discussion

In the present study the effect of prenatal stress by exposing dairy goats to heat during mating and early pregnancy was evaluated. We investigated whether gestational exposure to heat stress would have an impact on the behavior of the offspring later in life. Our HS pregnant goats experienced greater RT (+0.68°C) and RR (+76 breaths/min), but lower feed intake by 15% compared to TN goats. These findings indicate that HS treatment effectively triggered a significant heat stress response during pregnancy, which agrees with previous results obtained in goats exposed to HS conditions [24–26].

We hypothesized that heat stress during early gestation would influence both gestation and the development of the offspring postnatally. In this regard, the most relevant outcomes were the shortening of the gestation duration of HS goats by 3 days and the tendency of reduced of litter weight of IUHS kids compared to IUTN kids. Although in our study the association between the gestation length and the birth weight could not be confirmed, there is sufficient body of research that has confirmed this link in the past in sheep [27] and in cows [13,28]. These authors suggested that shorter time of gestation could lead to a reduction in the contributions of nutrients from the mother to the fetus. The last 2 months of the pregnancy period is when the greatest growth of the fetus occurs in cattle (60% of the weight at birth) [29], which could partially explain the lower litter weight of IUHS kids. In fact, both the shortening of the pregnancy and thus, the derived prematurity of the animals, and the thermal effect could be cofounded.

On the other hand, other authors [30] reported no effect of heat stress during the last 2 months of gestation on pregnancy length in cows, but birth weight of calves born from those heat-stressed cows is reduced. This finding suggests that the reduction in the duration of pregnancy itself is not the sole factor responsible for the decreased birth weight, and there may be other biological changes occurring during the *in utero* heat stress that affect birth weight. Heat stress during pregnancy is actually associated with poor placental development and lower blood flow, which may result in less nutrient flow to the fetus [29,31]. Additionally, Zhu et al. [16] reported that nutrient restriction of beef cows during the first third of gestation period results in reduced placental development and fetal weights. Hence, a reduction of nutrient supply during the first third of gestation (less feed intake by HS goats) could result in impaired placental function, which negatively affects growth during gestation and contributes to lower birth weight. Despite the observed differences in litter weight in the current study, body differences between IUHS and IUTN kids at 35 days of life were negligible, which indicates that IUHS kids were able to recover the loss of fetal body growth after birth.

In the present study we exposed IUTN and IUHS female kids to AT and NOT tests in order to assess their behavioral reactivity to a new environment and an unfamiliar object, respectively. The results indicated mild changes in the behavioral response of kids previously exposed to *in utero* heat stress. During the AT, IUHS kids showed a reduction in the number of sniffing events in the arena. When kids were exposed to NOT at 1 month of age, a reduction in exploratory behavior (i.e. sniffing events) was also confirmed, but these differences disappeared when kids were exposed again to NOT at 3 month of life. These results contrast with those obtained by Roussel et al. [21] who found that kids born to goats under transport stress explore the new environment (i.e. sniffing) more often than control kids. Some behavioral indicators such as immobilization, a reduction in explorative behavior, and reactivity towards humans have been related to fear [32–34]. At the hormonal level, these changes have been associated to alterations in the HPA axis [4] caused by an elevation of cortisol in the maternal circulating blood during the fetal development [21,35]. We did not measure blood cortisol levels in our pregnant goats or kids, but Hamzaoui et al. [24] reported that fecal corticosterone was not affected by chronic HS (i.e. 5 weeks of exposure to HS). It is also worth to mention that most of the development of the neural system takes place during the latter phases of gestation, and in our study, goats were not exposed to heat stress during late gestation. This could be a reason why the differences found in our animals were not similar to what reported in previous reports.

In a longer-term scenario, the effects of prenatal heat stress on kids were followed up in growing goats at 8 months of life. For that purpose, the behavior was assessed by scan-sampling before and after a heat challenge in order to elucidate whether *in utero* heat stress would have any effect on the response to HS in the postnatal life. Both IUTN and IUHS behaved similarly under TN and HS conditions. However, shifting from TN to HS resulted in significant behavioral changes to cope with the high ambient temperatures regardless the *in utero* thermal treatment. Resting and drinking increased dramatically during the first week of heat exposure. Lying and drinking behaviors could be considered as ideal biological markers for assessing the severity of the heat stress response [26]. Similarly, exploratory, grooming, and feeding behaviors declined throughout the entire period of heat exposure. Furthermore, rumination, an essential component of the ruminant behavior that is also used as an indicator of stress and anxiety [36,37], was reduced. These activities were also accompanied by changes in the posture of animals, spending longer time lying during heat exposure, and consequently shorter time was spent standing. Lying and inactivity are common behaviors observed during the exposure to high ambient temperatures as a strategy to reduce heat load. In addition, feeding behavior was decreased as a mechanism to reduce heat production [38,39].

Based on the behavioral changes observed in the current study, growing kid goats triggered an acute stress response during the first week of exposure to heat stress. However, the fact that lying and drinking were gradually decreased afterwards suggests that animals were able to progressively adapt to the rise of temperature. This ability to adapt to HS might be related to the breed used in the current study (i.e. Murciano-Granadina goats). Brown et al. [40] reported that the exposure of dairy goats to HS conditions (34°C and 25% humidity) depresses milk yield in Alpine but not in Nubian goats, indicating that the response to HS varies according to breed. Furthermore, lactating Murciano-Granadina dairy goats experience significant high rectal temperature and respiratory rate values under HS conditions, but these values are partially recovered to normal values after few days of heat exposure [11,24]. These findings reflect the high acclimation capacity of some goat breeds to hot conditions, which makes these breeds highly efficient under the expected climate change scenario.

In accordance with the results obtained in the AT and NOT, in which behavioral differences between IUTN and IUHS goats disappeared with age, most of the behavioral responses to the heat challenge at 8 months of age were also not affected by the *in utero* thermal treatment (no significant interaction between treatment and week). Only a tendency was observed for lying with straight legs (*P* = 0.099). Similarly, Akbarinejad et al. [41] could not demonstrate changes in the adaptation capacity after submission to heat stress occurring at first, second or last third of gestation in cows. Thence, although it seemed that kids were affected at birth and early age, results observed later in life would suggest that HS during early gestation would not affect the offspring behavior at long term. From the behavioral point of view, heat stress during the first third of gestation may not induce changes in the adaptive capacity of the offspring when exposed to heat challenge during the postnatal life.

## Conclusions

Heat stress during the period of mating until the first 45 days of gestation in dairy goats shortened the duration of pregnancy and tended to reduce the litter weight of kids. However, prenatal HS had no effect on kid morbidity or mortality. The behavioral response of kid goats to a novel environment and objects was altered by the *in utero* HS. The exposure of the fetus to a specific stress type (i.e., heat stress) can modify its ability to respond to other types of stress (e.g., environmental stress) in the early postnatal life. Nonetheless, under the conditions of this study (gestation stage at which dams were heat-stressed, HS intensity, and breed) such an impact disappeared towards the adult life of the animals with no differences in adaptability to heat stress. Regardless the *in utero* HS treatment, when Murciano-Granadina goats were exposed to heat challenge test at 8 months of age they experienced acute HS-related behavioral responses, but they were able to partially recover the normal behavior after few days. The use of climate-resilient breeds (e.g. Murciano-Granadina) would be beneficial given the fact that the negative impact of climate change will be greater in the future.

## Supporting information

S1 Fig Picture of the experimental facilities. (A) Capture of the recording for the arena test (AT). (B) Capture of the recording for the novel object test (NOT).
(TIF)

S1 Table List of behavioral and postural parameters recorded by scan-sampling during the heat-challenge experiment in the growing goats. These parameters are drawn from the Welfare Assessment Protocol for Goats [23].
(DOCX)
