# Supplementary figures and images for: Prenatal heat stress effects on gestation and postnatal behavior in kid goats
Source: PLoS One. 2020 Feb 10;15(2):e0220221. doi: 10.1371/journal.pone.0220221 (PMC7010273; doi:10.1371/journal.pone.0220221)

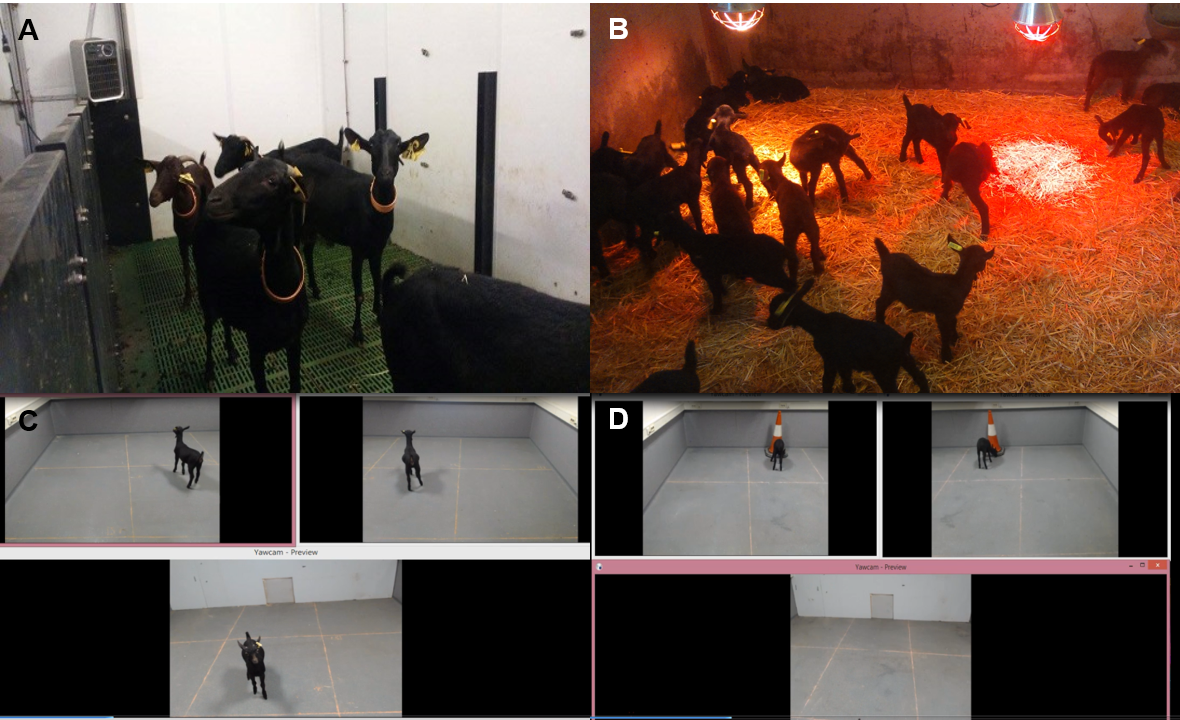

Supplement: S1 Fig — (A) Capture of the recording for the arena test (AT). (B) Capture of the recording for the novel object test (NOT). (TIF) [file pone.0220221.s001.tif]
