# Supplementary material for: Prenatal heat stress effects on gestation and postnatal behavior in kid goats
Source: PLoS One. 2020 Feb 10;15(2):e0220221. doi: 10.1371/journal.pone.0220221 (PMC7010273; doi:10.1371/journal.pone.0220221)
Supplement: S1 Table — These parameters are drawn from the Welfare Assessment Protocol for Goats [23]. (DOCX) [file pone.0220221.s002.docx]

**S1 Table. List of behavioral and postural parameters recorded by scan-sampling during the heat-challenge experiment in the growing goats.** These parameters are drawn from the Welfare Assessment Protocol for Goats [23].

|  | Description |
| --- | --- |
| Feeding behavior |  |
| Feeding | Head in the feeder |
| Rumination | Chewing rhythmically |
| Drinking | Mouth at the bucket of water |
| Non-feeding behaviors |  |
| Exploration | Sniffing the pen, feeder or the bucket of water |
| Grooming | Grooming itself |
| Other | None of the previous active behaviors nor resting |
| Resting |  |
| Thermally-associated behaviors | |
| Open-mouth panting | Accelerated respiration rate with open mouth |
| Closed-mouth panting | Accelerated respiration rate with close mouth |
| Postures |  |
| Standing-walking | Standing up displaying a no resting nor other behavior |
| Standing-immobile | Standing up displaying other active behavior |
| Lying-joint | Resting lying with legs drawn into the body |
| Lying-straight | Resting lying with legs held away from the body |
| Neck extended | The neck is extended on the floor |
